# Positive Emotions in Family Caregivers of Alzheimer’s Disease Patients: Factors Associated with Gain in Caregiving from a Gender Perspective

**DOI:** 10.3390/jcm13082322

**Published:** 2024-04-17

**Authors:** José Manuel Ponsoda, Amelia Díaz

**Affiliations:** 1 Faculty of Education, University of Alicante, 03080 Alicante, Spain; josemanuel.ponsoda@ua.es; 2 Faculty of Psychology, University of Valencia, 46010 Valencia, Spain

**Keywords:** gain, family caregivers, Alzheimer’s disease, gender, psychological distress, burden

## Abstract

**Background/Objectives**: Gender differences in the variables of burden, anxiety, depression, and others associated with psychological distress have been found in studies on caregivers caring for a dependent relative, but a gender perspective is seldom used when analysing the positive aspects of caregiving. This study contributes to filling this gap by analysing gender differences in caregivers in a specific positive variable: gain. **Methods**: A cross-sectional design was used in a sample of 44 male and 96 female caregivers from Family Alzheimer Associations. Gender differences were analysed in demographic and psychological variables associated with the caregiving situation. **Results**: Female caregivers showed higher psychological distress than male caregivers, but gender differences in gain were only obvious when a deeper analysis of the GAIN scale responses was performed. The mediational role of psychological distress and other predictive variables showed a different pattern in male and female caregivers. The important predictive and mediating role that psychological distress plays in the greater perception of gains in caregiving and the result showing that female caregivers are the ones with poorer mental health support the need for preventive and therapeutic programs specifically targeting the positive aspects of caregiving in female caregivers. **Conclusions**: Three aspects could be highlighted in this study: family caregivers of AD patients perceived gain in the caregiving situation; gender plays a differential role in the perception of gain; and, finally, psychological distress should be the target when interventions are planned, not only to alleviate negative aspects but also to increase the positive aspects of caregiving.

## 1. Introduction

Alzheimer’s Disease (AD) is the most common form of dementia, accounting for 60–70% of the more than 55 million cases of dementia in the last year worldwide [1]. Most people caring for these patients are relatives, family members or loved ones, friends, or neighbours, with a higher proportion of women than men [2]. Caring for a relative with Alzheimer’s disease is a difficult task. Depending on the phase of the disease the care recipient is in, the caregiver will face different situations. In the beginning, the person with AD will be able to keep their independence, performing almost all the basic and instrumental daily activities, including driving, shopping, and social interaction, but progressively, the first confusions, forgotten recent situations, disorientation, and difficulties making decisions appear. The caregiver, at this phase, must provide emotional support and supervise complex tasks. In the moderate phase, the cognitive and functional capacities are compromised, and the caregiver gives assistance in basic and instrumental activities and deals with personality and behaviour changes. In the most advanced and last phase of AD, when the capacities are more and more diminished, with language, movement, and communication severely limited, the relative with AD needs 24 h care [3].

The process of caring for a relative with dementia is different from caring for a dependent relative without dementia. The progressive loss of the loved one, when the care recipient no longer recognizes family members and, on the other hand, the caregiver does not perceive the psychological substance of the individual’s personality in the care recipient, even though the person physically remains the same, is one of the toughest parts of caregiving in AD. This is accompanied by anticipatory grief, described as one of the biggest barriers in the caregiver role of AD, representing one of the most relevant factors to consider in the last phases of AD, affecting 75% of the caregivers [4].

The care of dependent people in Spain is performed mostly by family members, and it takes the form of unpaid work carried out mainly by women [2]. In the specific case of caring for a relative with dementia, the picture is similar, with women being the primary caregivers in 85.25% of cases, usually a daughter between 50 and 60 years old, and providing 70% of the hours dedicated to caring [5]. Studies evaluating gender differences on the impact of caring for a relative with dementia have shown that women present worse mental health than men, with variables such as burden [6,7] or depressive and anxiety symptoms showing higher levels in women than in men [8,9]. These studies highlight the negative aspects associated with caregiving [10,11] and could predict that these caregivers would have a lower chance of experiencing positive emotions than other caregivers caring for a relative in a different health situation [12]. However, there is increasing evidence confirming that these caregivers also experience positive aspects, such as satisfaction with life, satisfaction with caregiving, gain, or optimism [13]. Additionally, there is evidence that these variables are very important in the day-to-day caregiver’s well-being [14]. Therefore, it seems necessary to study to what extent gender differences also occur in variables associated with positive aspects of caregiving.

Models and theories on positive aspects of caregiving have been present since the incorporation of positive psychological functioning and coping strategies in Lazarus and Folkman’s Transactional Model of Stress and Coping [15]. Years later, Folkman stated that both positive and negative emotions coexist as a response to stressful situations [16]. These positive emotions work as a coping strategy, a way to find positive aspects in adversity, guiding people to understand and accept what happens to them. Tennen & Affleck [17] proposed three theoretical assumptions of what they called “benefit-finding” in adverse, stressful, and threatening circumstances: (a) it is a selective appraisal that minimizes the victimization and targets the positive parts of the situation; (b) it is a coping strategy; and (c) it appears later in the adjustment process to adversity. Considering this last assumption, Fredrickson [18] proposed that in stressful circumstances, positive emotions over time will build adaptive and lasting resources. Fredrickson et al. [19], in the Broaden and Build theory, stated that positive emotions “(i) broaden people’s attention and thinking, (ii) undo lingering negative emotional arousal; (iii) fuel psychological resilience; (iv) build consequential personal resources; (v) trigger upward spirals towards greater well-being in the future; and (vi) seed human flourishing” (p. 1375).

### Gain in Caregiving

Between the end of the last century and the beginning of the current one, different works brought attention to the excessive number of studies focused on negative and psychopathological aspects of caregiving and the scarce number of studies cantered on positive aspects of caregiving [20,21]. Kramer, in 1997 [20], performed one of the first literature reviews on the positive aspects of caregiving, more specifically, in the “gain in the caregiving experience”. She then asked, “where are we? and what next?” Twenty-seven years later, we can affirm that since then, the benefits, gain, satisfaction, meaningfulness, uplifts, optimism, and many other positive variables have been studied in caregiving, completing the picture of the caregiving experience.

Focusing on the term “gain”, Kramer [20] defined it as any positive aspect, whether practical or emotional, that results from the task of caregiving. She proposed a conceptual model based on Lazarus and Folkman’s stress and coping model [15], where appraisals of both strain and gain produce positive or negative indicators that will influence the well-being of the caregiver. Sanders [22], in a qualitative study with caregivers of Alzheimer’s disease relatives affiliated with an Alzheimer’s Association, found three themes related to gain in their caregiving: (i) spiritual growth and faith, which is the most frequent in her sample; (ii) personal growth, producing changes in the life of the caregivers, with more patience, caring, and thankfulness for the opportunity to care for someone; and (iii) feelings of mastery based on personal accomplishments. The three types of gain were produced through the process of caregiving as a transformation from who they were prior to caregiving to who they become because of their role as a caregiver. Similarly, Netto et al. [23] presented three types of gain, summarizing the first two postulated by Sanders as one: (i) personal growth and feeling of mastery in the care skills, (ii) spiritual growth, and (iii) ability to increase relations with the care recipient and other dependent adults. Based precisely on these three dimensions, Yap et al. [24] created the GAIN scale (Gain in Alzheimer Care Instrument).

At this point, with a definition of gain and the distinct types of gain, we may wonder what produces gain in caregivers and what variables are associated with or are good predictors of gain. From different studies, gain in caregivers has been associated positively with lower education [20,25,26], older age [25], satisfaction with life [20,27], problem-solving coping strategies [20,28], low perceived burden, good mental health [27,28], good physical status, having social resources [20], a lower possibility of institutionalizing the care recipient [29], unemployed status, more than 3 years of caregiving, caregiving for at least the 60% of the time, daily contact with the care recipient, minimal or no financial difficulties, an advance stage of dementia in the care recipient, no behaviour problems in the care recipient, avoiding criticism as a management strategy, attending caregiving educational and support programs [28], more hours caregiving per day and being a caregiver for more time [30], religiosity [31,32], and character strengths of hope, gratitude, zest, teamwork, love, creativity, and curiosity [26]. Although gender is a relevant variable in caregiving, with most women caring for relatives [2] and, in many cases, experiencing the worst part of the caregiving experience, such as higher depression, anxiety, and burden levels than men [6,7,8,9,33,34,35,36], to our knowledge, no study has evaluated gender as a factor associated with differences in gain when caregiving.

Therefore, the aim of this work is to evaluate the perception of gain presented by a sample of family caregivers caring for a relative with AD and its relations to AD phase in the care recipient and the caregiver variables as age, marital status, educational level, relation with the care recipient, perceived physical health, objective burden in the form of months/years and hours per day caregiving, subjective burden in the form of perceived burden, and psychological distress from a gender differential perspective. Additionally, the percentages of caregivers in the response levels of the GAIN scale will be presented and analysed.

## 2. Materials and Methods

### 2.1. Participants, Design, and Procedure

The convenience sample of 140 caregivers came from Family of AD’s Associations in the Valencian Community in Spain; 96 were female, and 44 were male caregivers. The inclusion criteria were as follows: (1) the relative receiving care had been diagnosed with AD, (2) the relative with dementia was living in the community, and (3) there was no reading or understanding problem hindering the completion of the information requested. The AD diagnostic and specific AD stage classification by Feldman and Woodward [37] were completed by the neuropsychiatrist working in the corresponding health centre.

The study design is cross-sectional. All participants were recruited from Family AD’s Associations and assessed individually after obtaining the signed informed consent. The participation was voluntary, and no reward was given for the participation. To preserve the participants’ anonymity, a number was designated to the questionnaires and datasheet of each participant. The permission to perform this research was obtained from both the Family Alzheimer Associations and the Ethical Committee for Scientific Research of the University of Valencia (H1367489852167).

### 2.2. Measures

#### 2.2.1. Psychological Distress

The 12-item General Health Questionnaire, GHQ-12 [38], measured psychological distress. The response scale has a Likert format with four levels (0,1,2,3), where high scores indicate high psychological distress. This questionnaire is used in health centres as an initial screening mental health tool for measuring somatic or social dysfunction, anxiety, and depression symptoms [39]. Reliability measured as internal consistency in this study was adequate (Cronbach’s *α* was 0.87).

#### 2.2.2. Burden

The caregivers’ perceived burden was measured with Zarit’s Caregiver Burden Interview, CBI [40], with 22 items answered in a Likert scale format ranging between zero (never) and four (nearly always). High scores expressed a high perceived burden. Cronbach’s *α* was 0.85 in this study.

#### 2.2.3. Gain

Gain in caregiving was assessed with the GAIN scale (Gain in Alzheimer Care Instrument) developed by Yap et al. [24]. In this study, the Spanish version was used [26]. It is composed of 10 items with a Likert response scale of five levels from 0 = very disagree to 4 = very agree. The scale assesses gain or benefits related to the caregiving of a patient with AD. High scores indicate a higher level of perceived gains. The reliability obtained in this study using Cronbach’s α was adequate, and its value was 0.81.

#### 2.2.4. Sociodemographic Variables, Objective Burden, and Perceived Physical Health

Questions about sociodemographic variables such as age, marital status, educational level, relation with the care recipient, months/year in the caregiver role, and hours per day caring were included in a survey. Perceived physical health was measured with a single item, “how do you perceive your current physical health”, using a five-point scale: 5. Very good, 4. Good, 3. Neither good nor bad, 2. Bad, 1. Very bad.

### 2.3. Statistical Analysis

The first step in statistical analysis was to compare male and female caregivers in the categorical variables of age, education level, marital status, hours caring every day, perceived physical health, relation with the AD relative, and AD phase of the care recipient using the *χ*^2^-value. In the second step, the gender differences were calculated for the continuous variables of age, months in the caregiver role, perceived burden, and psychological distress through Student’s *t* and Cohen’s *d* tests. In a third step, the relationships between the variables studied and gain were calculated with Pearson’s *r* for continuous variables and Spearman’s rho for categorical variables in the whole sample, in male and female caregivers. In a fourth step, mediational analyses were performed to find the mediational role of psychological distress between perceived burden and gain in the whole sample, in male and female caregivers. Finally, we compared the response categories in the GAIN scale percentages between the original first study of Yap et al. [24] and our study, comparing those participants who perceived low gain in caregiving and those who perceived high levels of gain in their caregiving using the *χ*^2^-value and the Mann–Whitney’s U. All analyses were performed with the statistical software SPSS version 28, except for the mediation analyses in which case the PROCESS macro for SPSS [41] was used.

## 3. Results

### 3.1. Gender Differences

Table 1 shows the number, percentages, χ^2^-value, and significance of the demographic variables, education, marital status, relation with the AD patient, and patient AD phase in male and female caregivers. There were no significant gender differences in these variables. Most of the male caregivers have an education level of primary and secondary studies (40.9%), while most female caregivers have primary studies (46.9%). In addition, both male and female caregivers (85.4%) were mostly married. Over half of the caregivers were also sons (63.6%) or daughters (69.8%) of the AD patients. Additionally, almost half of male (45.5%) and half of female (50%) caregivers were caring for a relative in the moderate phase of AD. Finally, the age range was between 18 and 91 years old, and the average age was 55.89 years for the whole sample, 58.81 years for male and 54.55 years for female caregivers.

Table 2 shows the gender differences in the variables of age, months of caring, perceived burden, psychological distress, and gain. Only psychological distress showed significant differences, with female caregivers presenting more psychological distress than male caregivers, with a moderate size effect (*d* = 0.54). Female caregivers were younger and showed lower gain scores than male caregivers, both as a tendency (*p* = 0.067 and *p* = 0.077, respectively), but the differences were not significant.

### 3.2. Correlational Analysis

Table 3 shows the relationships between gain and the remaining variables. The pattern shown by the whole sample is similar to the one shown by the female caregivers, with negative and significant relations between gain and perceived burden and psychological distress and a positive and significant relation between gain and AD phase in the relative. Male caregivers presented a significant and positive relation between gain and months caring and hours per day caring, which can be assimilated to objective burden. However, male caregivers show a negative and significant relation between gain and marital status. Therefore, a higher perception of gain is present in female caregivers with lower perceived burden, psychological distress, and caring for a relative in a more advanced phase of AD, although in this case with a low value (0.19), while male caregivers perceived higher gain when they were not married (single, separated, and widowed), caring for more hours every day, and were a caregiver during more months or years.

### 3.3. Mediational Analysis

Due to the significant relationships between burden, psychological distress, and gain, three mediational analyses were performed for the whole sample and for male and female caregivers. The mediation analyses were based on ordinary least squares regression and the bootstrap method with 10,000 bootstrap samples. The statistical significance of the indirect mediating effects of variables upon the bootstrap method is evaluated based on whether the point estimate of the mediating variable is zero within a 95% bias-corrected and accelerated confidence interval (BCa CI).

**Figure 1:**
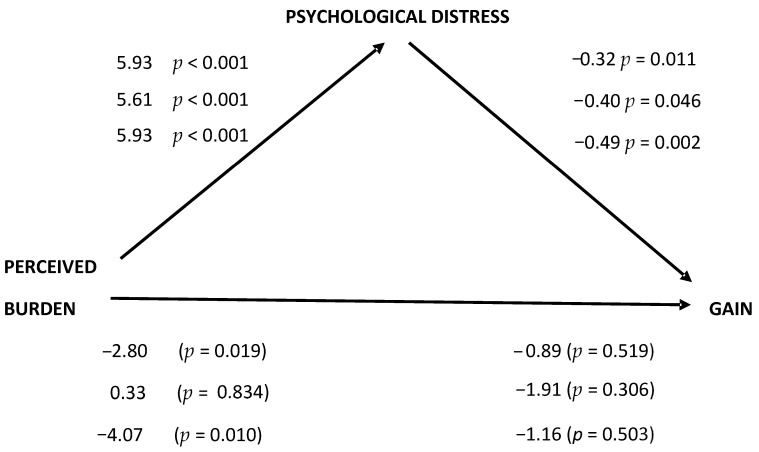
Mediation of psychological distress between perceived burden and gain. Unstandardized β values. First line corresponds to the whole sample, second line corresponds to male caregivers, and third line corresponds to female caregivers.

Figure 1 shows the unstandardized β values between the three variables. A full mediation was produced by the mediational role of psychological distress between perceived burden and gain for the whole sample and for female caregivers, but not for male caregivers. The significant unstandardized β between perceived burden and gain of −2.80 (*p* = 0.019) for the whole sample and −4.07 (*p* = 0.010) for female caregivers became the non-significant ones of −0.89 (*p* = 0.519) and −1.16 (*p* = 0.503), respectively, when psychological distress was introduced in the regression equation as a mediator variable.

Looking at the indirect effects of psychological distress between perceived burden and gain, shown in Table 4, all were significant in the whole sample and in the female and male caregiver subsamples due to the zero not being included between the lower and the upper bootstrapping 95% CI, where even for male caregivers, with no significant unstandardized β value between perceived burden and gain, the indirect mediational effect of psychological distress is significant.

### 3.4. Comparison of Studies and Gender Differences in the GAIN Scale Responses

The percentages of caregivers answering each category in the Likert format of the GAIN scale in this study are presented in Table 5. Two main aspects attracted our attention: the first was the higher percentages of caregivers choosing the “agree” categories, and the second was that the item referring to the contribution of caregiving to establish stronger bonds between the family members was the one with the lowest score. Therefore, caregivers perceive gains in caregiving in a high percentage, with a range of percentages from 56 to 84 of caregivers perceiving these gains.

The distribution of responses in the GAIN scale in our study allows knowing which caregivers are placed in the disagree levels (0 to 19 scores), with low gain, and who among them are in the agree levels, showing high or very high gain scores in caregiving (30 and 40 scores). The remaining participants were in the “neither agree nor disagree” level (20–29 scores). As we can see in Table 6, there is a significant gender difference in the distribution of responses in the GAIN scale (*χ*^2^ = 9.19; *p* = 0.010). It is worth noting that 12 (8.57%) participants were in the disagree levels, perceiving low levels of gain in caregiving, and 83 (59.3%) were in the agree levels, perceiving high to very high gain levels. The 12 participants perceiving low or no gain in caregiving were all female caregivers. In the intermediate level, “neither agree nor disagree”, there were more female caregivers (35.4%) than male caregivers (25%). Finally, more male caregivers (75%) than female caregivers (52.1%) were in the agree levels, showing a high gain in caregiving for an AD relative. These data show that, although the gender differences in Table 2 show no significant differences between male and female caregivers in the gain variable, a closer look shows clear gender differences in the perception of gain when caring for a relative with AD.

In the comparison between the 12 female caregivers in the low gain level with the 83 male and female caregivers in the high gain level, the variables of age and months of caring showed no significant differences, but psychological distress (low gain group: *M* = 18.83, *SD* = 7.60; high gain group: *M* = 13.74, *SD* = 5.04; Mann–Whitney’s *U* = 2.25; *p* = 0.043) and perceived burden (low gain group: *M* = 57.25, *SD* = 14.89; high gain group: *M* = 49.70, *SD* = 10.95; Mann–Whitney’s *U* = 2.13; *p* = 0.036) both presented significant differences. The comparison between the low- and high-gain groups in the categoric variables relation with the AD relative, relative’s AD stage, education, marital status, hours per day caring, and perceived physical health showed significant differences in the variable relation with the AD relative (*χ*^2^ = 15.88; *p* = 0.007) and caregiver perceived physical health (*χ*^2^ = 21.06; *p* < 0.001). A higher percentage of sons and daughters of the AD relative, and caregivers with a better perception of their physical health were in the high gain group.

## 4. Discussion

For a long time, research on family caregivers of AD patients traditionally showed only the negative aspects, the symptoms of the heavy burden supported by caregivers [4,10,11]. However, in the last few decades, an important amount of research has been performed looking for the positive aspects of caregiving. When Kramer presented, in 1997 [20], the first revision of positive emotions in the caregiving situation, a wide door was open in the study of benefits, satisfaction, gain, and many other positive variables, framed by theories specifically thought and adapted to the caregiving experience [17,20,22,23]. The present work is a step in this direction, the study of gain associated with the caregiving experience from a gender perspective.

Firstly, our sample, containing more female than male caregivers, as is usual when caring for a relative [2], presented no significant gender differences in most of the variables assessed, including similar age, education, marital status, hours per day and months of caring, relation with the AD relative and phase of the AD relative, perceived burden, and gain. Only psychological distress showed significant differences, with female caregivers scoring higher than male caregivers. Worse mental health status in female caregivers than in male caregivers is also a usual finding in this field of research [8,9]. Although this result confirmed several previous ones, one of the aims of our study was to find out if female caregivers also scored lower in the perception of gain when caregiving.

When the perception of gain in male and female caregivers’ means was compared, male caregivers obtained a mean of gain higher (32.0) than female caregivers (29.99), but the difference was not significant, showing just a tendency. However, a closer look at the responses to the GAIN’s items gives us a clearer pattern. The lower categories in the response format, with the lowest level of gain, were only selected by 12 participants, all female caregivers, whilst the high gain level categories of the scale had a higher representation of male caregivers (75%) than female caregivers (52.1%), showing significant gender differences in the comparison of percentages. These gender differences between high and low levels of gain reflect the higher perceived burden and mental health problems, together with the more distant relationship with the AD care recipient of the female caregivers at the lower level compared to the male and female caregivers at the higher level. The latter were represented more by sons and daughters of the family relative with AD and a better perception of physical health status than those female caregivers in the low levels of gain. These results fill a gap in the study of gender differences in the research of gain in caregiving and show that a deeper analysis is necessary to have a complete picture of the issue. Although there is a research work studying differences in the positive aspects of caregiving between spousal caregivers [42], to our knowledge, this is the first study that analysed gender differences by having a closer look into the GAIN responses categories.

Our work clearly shows gain in caregivers of AD relatives, with a mean of over 30 on the GAIN scale, where the maximum score is 40 in the whole sample. Different studies, both in Spain and in other countries, confirmed that the average score on the GAIN scale is around 30 [24,26,28,29,30,31]. The GAIN scale was introduced by Yap et al. in 2010 [24] using a sample of AD caregivers in Singapore. If we compare our results with the results of this original study, a similar pattern in the percentages of caregivers in each response category in the scale becomes noticeable. This stability over time and cultures seems to be present in the measure of gain since the GAIN scale was published. Parveen and Morrison [30] also found stability in the variable gain over a time of 9 months in a longitudinal study in a sample of family caregivers in the UK. Our results also confirm this stability. Another result provided further confirmation since the first publication of the GAIN scale concerns the specific item with the lowest score, the one that asks if the care for a relative helped to strengthen familial bonds [24,31]. It seems that caring for a relative with AD could create family conflict when the resources and social support are insufficient [43].

Our study shows that predictors of gain in caregiving should consider the gender of the caregiver. Looking at the relationship between gain and other variables, caring for a relative in a more severe AD phase and caregivers’ perception of low burden and psychological distress are good predictors of gain, especially in female caregivers, while more hours caring every day, more months and years caring for the AD relative, and a non-married marital status are also good predictors for male caregivers. We also found that being sons or daughters of a relative with AD and perceiving a good physical status were good predictors of perceived gain in caregivers of both genders. Previous studies found predictors of gain in low perceived burden and good mental health in caregivers [27,28], caregiving for more than 3 years and at least 60% of the time [28], and good physical status [20], results that are confirmed in this study. However, to our knowledge, no previous study showed the predictive role of being the son or daughter of the care recipient in the gain variable. The gender analysis performed showed different predictors of gain for male and female caregivers, although when the whole sample is considered, there is a coincidence with those of female caregivers’ predictors but not with those of male caregivers, hence, the relevance of a gendered approach. The specific predictive role of months and years of caregiving in male caregivers and caring for relatives in an AD advanced stage in female caregivers support the assumption that to perceive gain in caregiving, it is necessary for the caregiver to adapt to the adverse situation [17], allowing positive emotions over time to build adaptive and lasting resources [18] and triggering upward spirals towards greater well-being in the future [19].

Psychological distress should be highlighted both for the predictor role of gain shown [28] and for the mediational role presented between perceived burden and the perception of gain in caregivers of AD relatives. Basically, when psychological distress is low, the negative action of the perceived burden on gain is lost, with caregivers perceiving gain even in the circumstance of a high perceived burden. This process is applicable to both female caregivers and when we consider the whole sample, but in the specific case of male caregivers, although psychological distress plays a mediational role between perceived burden and gain, the lack of prediction of perceived burden on gain makes the process less accurate. Wong et al. [42] found a similar result studying Chinese spousal caregivers where positive aspects of caregiving produced a moderating effect between perceived burden and depression, anxiety, and overall psychological distress for wife-caregivers but not for husband-caregivers, although, as in our study, female caregivers reported lower positive aspects of caregiving, higher psychological distress, and perceived burden. The relevance of psychological distress in female caregivers is of vital importance if the objective is to increase gain in their caregiving role. Although both male and female caregivers could present positive and negative aspects associated with caregiving [13,14], the perception of gain in female caregivers is much more dependent on their mental health, and consequently, this highlights the need for a gendered approach.

This study has several limitations. Firstly, having a cross-sectional design, no cause–effect inferences can be made. Secondly, the sample is a convenience one, so as a non-random and probabilistic sample, the generalization of the results should be performed carefully and only if the sample characteristics are similar. Thirdly, the variables were assessed with self-reports, which gives the caregivers’ perception of their specific situation but are also subject to different biases such as social desirability. On the other hand, this work represents an advance in the evaluation of positive emotions in caregivers, confirming that caregivers of people with AD present both burden and mental health problems, as well as positive emotions like gain in caregiving. It should be noted that the proportion of caregivers with a perception of gain in caring is high. The comparison of our data with those obtained in Singapore fourteen years ago shows that even two different and distant cultures are similar in the way they perceive gains in caring for a relative with dementia. The gender perspective taken in this work reveals that in positive emotions as well, the lowest levels are seen in female caregivers. If we add to this the important mediating role that psychological distress plays in the greater perception of gains in caregiving and that female caregivers are precisely the ones who have poorer mental health, support, preventive, and therapeutic programs targeting PAC in female caregivers become necessary.

## Figures and Tables

**Table 1:** Demographic characteristics, hours per day caring, perceived physical health and relation, and AD stage of the relative in the male and female caregivers.

	Male		Female		
Variables	*n*	%	*n*	%	*χ*^2^-Value (*p*)
Education level					2.25 (0.522)
No studies	3	6.8	3	3.1	
Primary	18	40.9	45	46.9	
Secondary	18	40.9	32	33.3	
University	5	11.4	16	16.7	
Marital Status					0.30 (0.587)
Single/separated/widow	8	14.6	14	14.6	
Married	36	85.4	82	85.4	
Hours/day caring					2.18 (0.535)
<5 h	7	15.9	19	19.8	
5–10 h	8	18.2	26	27.1	
11–15 h	6	13.6	12	12.5	
>15 h	23	52.3	39	40.6	
Perceived physical health					6.87 (0.143)
Very bad	0	0.0	1	1.0	
Bad	2	4.5	4	4.2	
Medium	9	20.5	37	38.5	
Good	31	70.7	46	47.9	
Very good	2	4.5	8	8.3	
Relation with the AD patient					3.98 (0.552)
Espouse	11	25.0	13	13.5	
Daughter/son	28	63.6	67	69.8	
Daughter/son-in-law	3	6.8	10	10.4	
Grandchild	1	2.3	4	4.2	
Nephew/niece	1	2.3	2	2.0	
AD phase of the patient					0.25 (0.883)
Mild	14	31.8	28	29.2	
Moderate	20	45.5	48	50.0	
Severe	10	22.7	20	20.8	

**Table 2:** Gender differences in the variables age, months of caring, perceived burden, psychological distress, and gain.

	Male (*n* = 44)		Female (*n* = 96)				
	M	SD	M	SD	*t*	*p*	*d*
Age	58.81	13.90	54.55	10.95	1.80	0.077	---
Months caring	49.43	31.72	52.48	36.56	0.48	0.634	---
Perceived burden	50.25	10.53	52.10	13.03	−0.83	0.409	---
Psychological Distress	13.34	4.79	15.46	5.75	−2.13	0.035	0.54
Gain	32.02	5.04	29.99	7.84	−1.84	0.067	---

**Table 3:** Relationships between gain and the remaining variables.

	Variables	Whole Sample	Male	Female
Pearson’s *r* (*p*)	Age	−0.05 (0.534)	0.17 (0.274)	0.17 (0.094)
	Months caring	0.14 (0.111)	0.37 (0.013)	0.09 (0.410)
	Burden	−0.24 (0.005)	0.10 (0.658)	−0.30 (0.003)
	Psychological distress	−0.28 (<0.001)	0.27 (0.081)	−0.40 (<0.001)
Spearman’s *Rho* (*p*)	Studies	−0.06 (0.473)	−0.28 (0.069)	0.03 (0.775)
	Marital status	−0.03 (0.749)	−0.30 (0.049)	0.08 (0.420)
	Hours/day caring	0.10 (0.248)	0.32 (0.035)	0.07 (0.964)
	Perceived Physical Health	0.00 (0.962)	−0.25 (0.090)	0.10 (0.518)
	Relation with the AD patient	0.02 (0.815)	−0.10 (0.534)	0.07 (0.346)
	AD phase of the patient	0.19 (0.023)	0.16 (0.301)	0.23 (0.025)

**Table 4:** Indirect effects of perceived burden on perceived gain through psychological distress.

	Point Estimate	Standard Error	Bootstrapping 95% Confidence Interval	
			Lower	Upper
Whole sample	−1.91	0.94	−3.84	−0.12
Female	−2.91	1.12	−5.27	−0.82
Male	2.23	1.56	0.19	6.24

Whole sample *n* = 140; male *n* = 44; female *n* = 96; *K* = 10,000 bootstrap samples.

**Table 5:** Percentage of participants in each Likert format response level in this study.

	Disagree a Lot Score 0	Disagree a Little Score 1	Neither Agree Nor Disagree Score 2	Agree a Little Score 3	Agree a Lot Score 4	Mean Item Score
1. … be more patient and understanding…	6.4	2.9	8.6	32.1	50	3.16
2. … stronger and more resilient…	2.1	6.4	13.6	32.9	45.0	3.12
3. … more aware of myself…	0.7	5.0	20.7	35.0	38.6	3.06
4. … knowledge and skills in dementia…	0.7	4.3	10.7	29.3	55.0	3.34
5. … grow closer to my relative…	2.1	5.0	11.4	22.1	59.3	3.31
6. … bond my family closer…	2.9	9.3	25.0	30.0	32.9	2.81
7. … better relation to dementia and olders…	2.9	4.3	19.3	28.6	45.0	3.09
8. … insight into the meaning of life…	2.1	4.3	14.3	30.7	48.6	3.19
9. … grow spiritually…	13.6	10.0	20.0	27.9	28.6	3.48
10. … altruistic goals…	1.4	2.9	22.1	34.3	39.3	3.07

**Table 6:** Gender differences in the distribution of responses in the GAIN scale levels.

		Gain Scores							
		0–19		20–30		31–40			
		*n*	%	*n*	%	*n*	%	*χ*^2^-Value	*p*
Gender								9.19	0.010
	Male	0	0.0	11	25	33	75		
	Female	12	12.5	34	35.4	50	52.1		

## Data Availability

The datasets generated and analysed during the current study are available from the corresponding author upon reasonable request.
